# Synthetic versus natural curcumin: bioequivalence in an *in vitro* oral mucositis model

**DOI:** 10.1186/1472-6882-14-53

**Published:** 2014-02-11

**Authors:** Sonja C Lüer, Jeannette Goette, Rolf Troller, Christoph Aebi

**Affiliations:** 1 Division of Pediatric Hematology/Oncology, Department of Pediatrics, University of Bern, Inselspital, Bern CH-3010, Switzerland; 2 Institute of Hospital Pharmacy, University of Bern, Inselspital, Bern CH-3010, Switzerland; 3 Institute for Infectious Diseases, University of Bern, Inselspital, Bern CH-3010, Switzerland

**Keywords:** Curcumin, Synthetic, Bioequivalence, Mucositis, Cancer, Chemotherapy

## Abstract

**Background:**

Curcumin (CUR) is a dietary spice and food colorant (E100). Its potent anti-inflammatory activity by inhibiting the activation of Nuclear Factor-κB is well established.

**Methods:**

The aim of this study was to compare natural purified CUR (nCUR) with synthetically manufactured CUR (sCUR) with respect to their capacity to inhibit detrimental effects in an *in vitro* model of oral mucositis. The hypothesis was to demonstrate bioequivalence of nCUR and sCUR.

**Results:**

The purity of sCUR was HPLC-confirmed. Adherence and invasion assays for bacteria to human pharyngeal epithelial cells demonstrated equivalence of nCUR and sCUR. Standard assays also demonstrated an identical inhibitory effect on pro-inflammatory cytokine/chemokine secretion (e.g., interleukin-8, interleukin-6) by Detroit pharyngeal cells exposed to bacterial stimuli. There was bioequivalence of sCUR and nCUR with respect to their antibacterial effects against various pharyngeal species.

**Conclusion:**

nCUR and sCUR are equipotent in *in vitro* assays mimicking aspects of oral mucositis. The advantages of sCUR include that it is odorless and tasteless, more easily soluble in DMSO, and that it is a single, highly purified molecule, lacking the batch-to-batch variation of CUR content in nCUR. sCUR is a promising agent for the development of an oral anti-mucositis agent.

## Background

Curcumin (CUR), a natural compound extracted from the yellow root plant Curcuma longa (Linn.) [1], is a widely known spice and yellow colorant (E100) used in food and drinks worldwide [2]. Since millennia, CUR has also been highly appreciated as a medical remedy with anti-inflammatory and antimicrobial properties. Its therapeutic use in traditional medicines varies from topical application on wounds to systemic oral application [3]. Modern medicine recently became increasingly interested in the various biological effects of the active compounds extracted from the yellow root, mainly CUR and its derivatives. To test the effects of CUR in various settings in the laboratory, commercially available extracts from the root are used. The root itself contains about 3% of CUR (diferuloylmethane), several of its derivatives, and oils, resins and other co-factors from the rhizome. Thus, effects attributable to CUR as the active agent remain unclear in most experiments. Standardization of the effects of CUR and of curcuminoids remains difficult, since in the majority of studies the exact composition of the CUR powder used is not specified, and in preparations with a declared percentage of CUR content the remaining components are not outlined in detail. Thus, the effects seen could possibly be attributed in part to the combination of CUR and curcuminoids as well as to other components of the preparation. Most production processes include purification or concentration during or after the extraction from the root to achieve high concentrations of CUR. Even though standardized and regulated by authorities, there is a possibility of contamination of nCUR with substances used during plant growth, from the environment, and from the production and purification processes (e.g. fertilizers, heavy metals, spores).

To compare a synthetically manufactured high purity curcumin (sCUR) to a standard nCUR, we used our previously established *in vitro* mucositis system [4,5] and tested cytotoxic, anti-inflammatory, and antimicrobial properties of both substances to determine how effective the > 99% pure sCUR acts in comparison with standard nCUR. sCUR could possibly serve as a topical agent against cancer therapy-induced and other forms of oral mucositis.

## Methods

### Microorganisms, cell lines and culture conditions

Microorganisms were grown in Brain-Heart Infusion broth (BHI) at 37°C in a 5% CO_2_ atmosphere or in air at 150 revolutions per minute (rpm). Clinical isolates included *Moraxella catarrhalis* ATCC 25238 [4], *M. catarrhalis* O35E [6], *Streptococcus pneumoniae* serotype 6B [5] and nontypable *Haemophilus influenzae*[5]. The human pharyngeal cell line Detroit 562 (ATCC CCL 138) was maintained in Eagle’s minimal essential medium (MEM, Invitrogen, Basel, Switzerland) supplemented as indicated with heat-inactivated fetal calf serum (FCS), 2 mM L-glutamine, 1 mM sodium pyruvate (Sigma, St. Louis, MO), 1x nonessential amino acids (Sigma), 100 U/ml penicillin, and 100 μg/ml streptomycin at 37°C in an atmosphere containing 5% CO_2_.

### Reagents

nCUR, purified from *Curcuma longa* (Turmeric), was purchased from Sigma (St. Louis, MO) (No. C1386). According to the manufacturer, it contains > 65-70% diferuloylmethane (CUR) and greater than 90% curcuminoids by HPLC. Commercially available sCUR was obtained from Aptuit Laurus Ltd., Visakhapatnam, India, a Good Manufacturing Practice (GMP) approved manufacturing site. The procedure of synthesis is proprietary. The batches of sCUR used were reported to be greater than 99% pure by high pressure liquid chromatography (HPLC) and to contain residual amounts of ethyl acetate, methanol, toluene, and n-butanol. sCUR from this manufacturer fulfils FDA approved GRAS (Generally Regarded As Safe) safety criteria. We confirmed the purity of sCUR using our in house HPLC. As indicated by the manufacturer (purity, 99.6%), we found an overall purity of 99.7% (98.2% and 1.5% in keto- and enol-form, respectively). For use in experimental procedures, both nCUR and sCUR were solubilized in fresh dimethylsulfoxide (DMSO) (stock solution, 73.678 mg/ml, i.e., 200 mM) and added to cell culture or growth media. Thus, the standard working concentration of 200 μM CUR contained 0.1% DMSO. Also, negative controls (phosphate-buffered saline (PBS), MEM, BHI broth) contained 0.1% DMSO, unless noted otherwise.

### Detection of cytotoxicity

Cytotoxicity Detection Kit Plus (LDH)® from Roche Diagnostics GmbH, Mannheim, Germany was used to detect CUR-induced cytotoxicity to Detroit epithelial cells after 4 hours of exposure to various concentrations of CUR. Three independent experiments were performed.

### Time-kill experiments of bacteria exposed to CUR

*M. catarrhalis*, *S. pneumoniae* and nontypable *Haemophilus influenzae* were grown in BHI to an OD_600_ of 0.4 (~5×10^7^ colony forming units (cfu)/ml), aliquoted, and subsequently grown in medium supplemented with 20, 50, and 100 μM nCUR or sCUR, respectively. Growth in BHI containing 0.05% DMSO was used as control. Quantitative cultures were obtained by serial plating of 100 μl-aliquots at 0, 60, 120, 180 and 240 minutes, respectively.

### Epithelial cell adherence assays

The ability of nCUR or sCUR to inhibit the attachment of *M. catarrhalis* to Detroit cells *in vitro* was measured as previously described [7]. Briefly, Detroit cells (~2.5×10^5^ per well) grown to a confluent monolayer in 24-well tissue culture plates were exposed to various concentrations of nCUR or sCUR (0–200 μM) for 60 minutes in MEM supplemented with 10% FCS, followed by washing three times in MEM. Bacteria grown overnight were adjusted to a multiplicity of infection (MOI) of 30. Bacteria were then added to tissue culture wells in MEM without antibiotics, centrifuged for 5 min at 1500 rpm, and incubated for 30 minutes at 37°C. Wells were then washed 5 times in MEM, trypsinized, and the suspensions were cultured quantitatively to determine the number of adherent bacteria. Data were expressed as the proportion of bacteria, i.e. cfu, of the original inoculum adhering to the epithelial cells. Each assay was performed in triplicate and at least three experiments were performed.

### Epithelial cell invasion assays

Bacterial invasion was estimated using a conventional gentamicin protection assay as previously described [8] with the following modifications. Cells were prepared in MEM without antibiotics and subsequently exposed to CUR as described for the adherence assays. After washing, bacteria were added at MOI 30, centrifuged for 5 min at 1500 rpm and incubated for 3 h at 37°C in 5% CO_2_. To determine the number of intracellular bacteria, the infected monolayer was washed three times in PBS and treated with gentamicin sulfate (200 μg/ml) for 2 h at 37°C in order to kill all extracellular bacteria. After washing, cells were detached from the plastic surface by treatment with 0.25% trypsin-EDTA, lysed by the addition of 1% saponin, and serially diluted in PBS for quantitative bacterial culture. Invasion ratios were calculated by dividing the number of cfu recovered after gentamicin exposure by the number of cfu inoculated.

### Determination of cytokine/chemokine secretion by Detroit cells stimulated with live whole bacteria

Monolayers of Detroit cells in 24-well plates were prepared as described above and pre-incubated with nCUR or sCUR at 0 (i.e., MEM with 10% FCS and DMSO only), 100 or 200 μM for 0, 15, 30 or 60 min, respectively. Wells were then washed to remove CUR and subsequently infected at a MOI of 100. To exclude pro-inflammatory activation by CUR only, mock infection (MOI 0) after pre-exposure with 200 μM of CUR was performed as a negative control. Ten μg/ml of lipopolysaccharide from *Salmonella enteritidis* (Sigma) was used as positive control. After 4 hours, cell culture supernatants were removed, centrifuged and stored at -80°C before determination of cytokine/chemokine concentrations. Interleukin (IL)-8 was determined using a commercially available ELISA kit according to the manufacturer’s protocol (R&D Systems, Minneapolis, MN). For determination of additional cytokines and chemokines in the same cell supernatants the Luminex® xMAP® technology was used [5]. Milliplex map kits (Milliplex Corporation) were used for microsphere-based multiplex immunoassays. Using a commercially available eight-plex kit, IL-6, IL-8, IL-10, Monocyte chemoattractant protein 1 (MCP-1), Tumor Necrosis Factor α (TNF α), Vascular Endothelial Growth Factor (VEGF), Fibroblast Growth Factor 2 (FGF-2), and Granulocyte Macrophage-Colony Stimulating Factor (GM-CSF) were determined according to the manufacturer’s instructions. Supernatants from three independent experiments, each run in duplicates, were tested. Values below the detection limit were given an arbitrary value suggested by the manufacturer.

### Statistical analysis

Two-way analysis of variance (ANOVA) with Bonferroni’s post-test correction was used (GraphPad Prism 5.02 statistics package, San Francisco, CA). P values < 0.05 were considered statistically significant.

## Results

### Characterization of nCUR and sCUR

**Figure 1:**
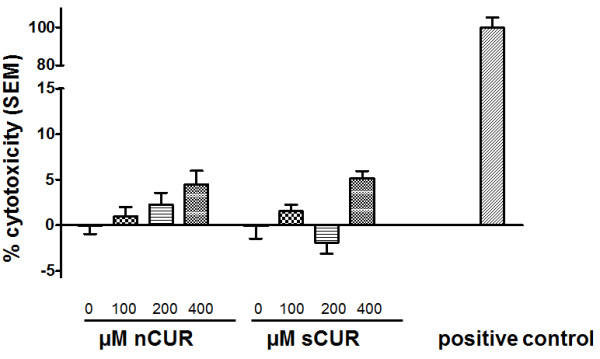
**Cytotoxicity of CUR for Detroit 562 cells.** Cytotoxicity assay. Monolayers of Detroit 562 cells were exposed to various concentrations of nCUR or sCUR, respectively, as indicated. The proportion of toxicity is indicated as mean + 1 SEM of three consecutive experiments run in triplicate, i.e., 9 data points per condition were tested. The positive control was provided by the manufacturer of the kit and led to complete cell lysis (defined as 100% lysis).

While nCUR is a yellow powder, which at our standard working concentration of 200 μM has its characteristic smell and musky, bitter flavor, sCUR, an orange-colored powder, is entirely odorless and tasteless. Maximum solubility in water, PBS or MEM supplemented each with 0.1% vol/vol of DMSO was somewhat better for sCUR at 500 μM. Both nCUR and sCUR demonstrated minimal and comparable cytotoxicity when LDH was determined in the supernatant after a 4-hour exposure of a monolayer of Detroit cells to up to 400 μM CUR (Figure 1). It is of note that at concentrations exceeding 200 μM nCUR was not entirely in solution. Although the fluid appeared clear, centrifugation resulted in the formation of a small pellet.

### Bactericidal activity of nCUR versus sCUR

**Figure 2:**
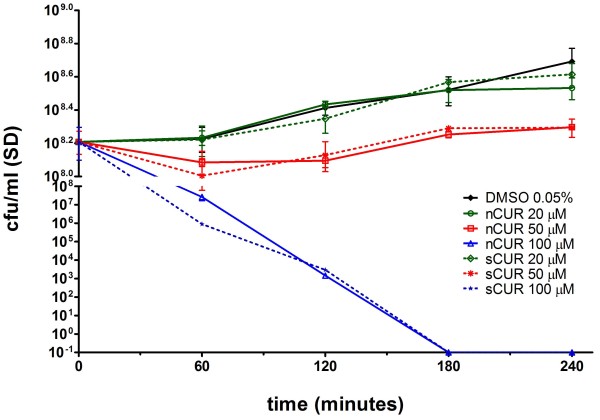
**Bactericidal effects of nCUR versus sCUR.** Comparative quantitative time-kill analysis of nCUR versus sCUR for *M. catarrhalis* strain O35E. Quantitative cultures were obtained at 0, 60, 120, 180, and 240 minutes. Killing kinetics were similar for both nCUR and sCUR at all concentrations tested (20, 50, and 100 μM). The negative control medium consisted of BHI containing 0.05% DMSO.

CUR is known to have antibacterial and antifungal activity, the extent of which is both species- and strain-dependent [5]. In order to compare nCUR and sCUR we chose two well characterized strains of *M. catarrhalis* for time-kill analyses. While strain ATCC 25238 was highly susceptible to CUR (data not shown) [5], a concentration of 100 μM CUR was required to result in a bactericidal effect in strain O35E (Figure 2). Importantly, there was no difference between nCUR and sCUR. *S. pneumoniae* serotype 6B and non-typable *H. influenza*e were also tested with no difference between nCUR and sCUR (data not shown).

### Inhibition of bacterial adherence and invasion to epithelial cells

**Figure 3:**
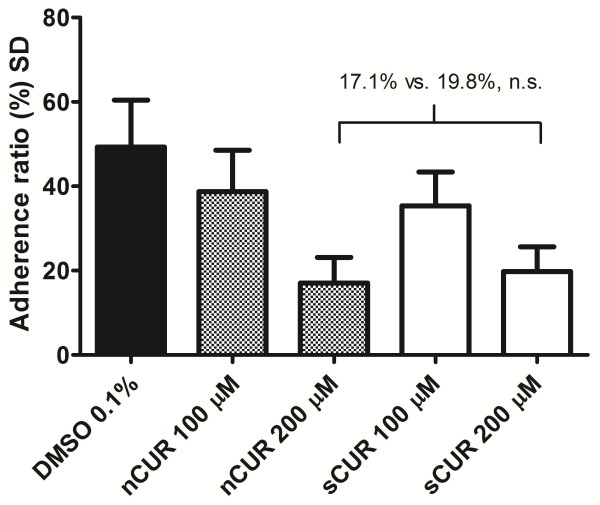
**Effect of nCUR versus sCUR on bacterial adherence.***M. catarrhalis* adherence assay. After pre-incubation of Detroit cells for 60 minutes with nCUR or sCUR, respectively, at the indicated concentrations, bacteria (*M. catarrhalis* ATCC 25238) were inoculated onto the monolayers at a MOI of 30, centrifuged, and incubated for 30 min at 37°C. Non-adherent bacteria were removed and adherent bacteria were determined by quantitative culture of trypsinized cells. The overall ANOVA test statistic revealed a p < 0.0001, the between-column values calculated using Bonferroni’s multiple comparisons test were >0.05 for nCUR versus sCUR at 100 or 200 μM, respectively. Mean adherence ratios + 1 SD of three independent experiments, each run in triplicate, are shown. MEM containing 0.1% DMSO, but no CUR, was used as control.

**Figure 4:**
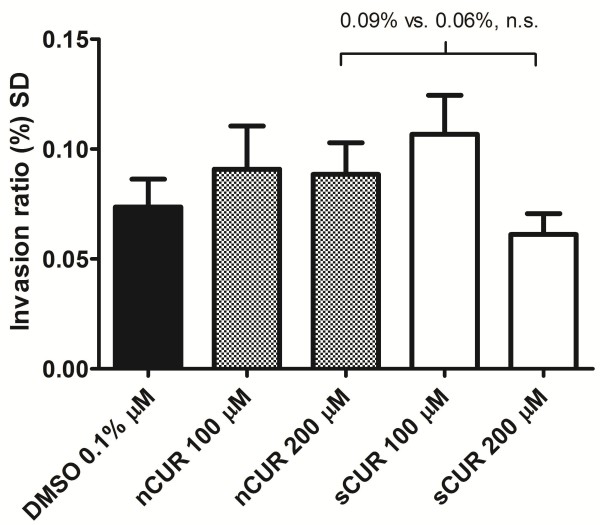
**Effect of nCUR versus sCUR on bacterial invasion.** Determination of invasion of Detroit cells by *M. catarrhalis* strain 25238 using a gentamicin protection assay. Cells were pre-treated with 0, 100 or 200 μM of nCUR or sCUR, respectively, for 1 hour. Following 3 hours of infection, extracellular bacteria were killed by exposure to gentamicin for 2 hours. Following cell lysis, quantitative bacterial cultures identified viable intracellular bacteria. The ANOVA overall p value was 0.112. In-between column p values were > 0.05. Mean adherence ratios + 1 SEM of three independent experiments, each run in triplicate, are shown.

While 100 μM CUR did not significantly inhibit bacterial adherence to Detroit cells (Figure 3), both 200 μM nCUR and sCUR demonstrated a statistically significant inhibitory effect. There was, however, no difference between the two forms of CUR (nCUR, 17.1% vs. sCUR, 19.8%, not significant). Both values were significantly lower than the control (49.8%, p < 0.05). The effect of CUR on cellular invasion of bacteria using the gentamicin protection assay was more difficult to assess. In contrast to previous findings [4], CUR did not significantly inhibit invasion. Although the proportion of invading bacteria was somewhat lower in cells exposed to 200 μM sCUR, this difference was not statistically significant (Figure 4).

### Inhibition of epithelial cytokine/chemokine release by CUR

**Figure 5:**
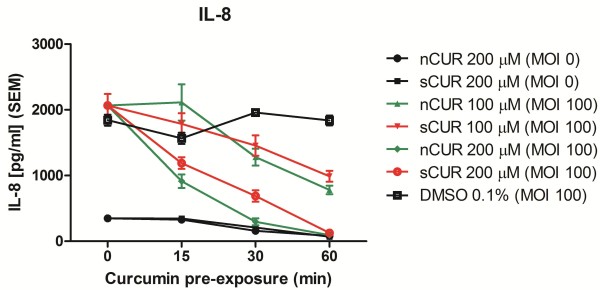
**Inhibition of IL-8 secretion by nCUR versus sCUR.** IL-8 secretion by Detroit cells preincubated for 0, 15, 30 or 60 minutes with nCUR (green) or sCUR (red) and subsequently infected for 4 hours with live *M. catarrhalis* strain 25238 (MOI 100). □ indicates the positive control (0 μM CUR, MOI 100). ● (200 μM nCUR, MOI 0) and ■ (200 μM sCUR, MOI 0) are negative controls. At no time point there is was a statistically significant difference between nCUR and sCUR for a given concentration.

**Figure 6:**
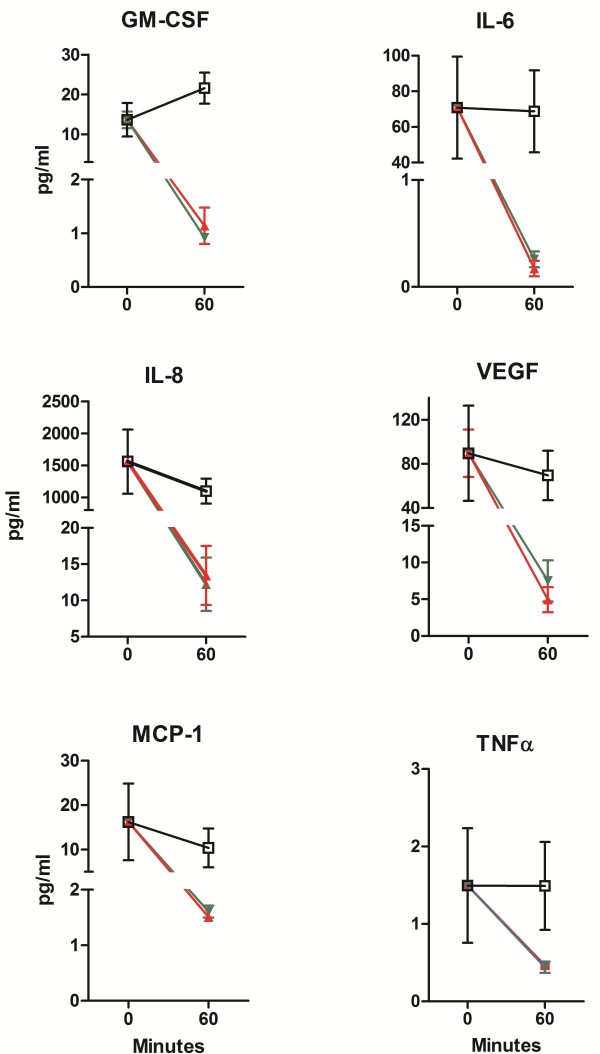
**Inhibition of release of pro-inflammatory cytokines by nCUR versus sCUR.** Effect of nCUR and sCUR on secretion of GM-CSF, IL-6, IL-8, MCP-1, VEGF, and TNFα by Detroit cells preincubated for 0 or 60 minutes with 200 μM nCUR (green) or 200 μM sCUR (red) and subsequently infected for 4 hours with live *M. catarrhalis* strain 25238 (MOI 100). □ (black) indicates the positive control (0 μM CUR, MOI 100). At no time point there is a statistically significant difference between nCUR and sCUR.

As previously established, IL-8 was used as a representative of pro-inflammatory cytokines/chemokines [4,5]. Figure 5 demonstrates that both nCUR and sCUR similarly inhibit IL-8 secretion by Detroit cells (no statistical difference). This effect was seen for both concentrations of CUR tested. In addition, Luminex technology was used to assess the same cell supernatants for concentrations of GM-CSF, IL-6, IL-8, IL-10, MCP-1, VEGF, FGF-2, and TNFα. As expected [5], the release of all cytokines/chemokines with the exception of IL-10 and FGF-2 (not shown) was fully suppressed by pre-exposure to nCUR or sCUR for 60 minutes followed by stimulation of the monolayer with a bacterial MOI of 100 for 4 hours (Figure 6).

## Discussion

As of today, *in vitro* laboratory work and clinical trials with CUR (diferuloylmethane) have exclusively used nCUR purified from the root of *Curcuma longa*[9] or chemically synthesized derivatives or nanoparticles [10,11]. This is a reasonable approach because highly purified nCUR can be produced in large quantities and has proved effective in many experimental assays and as a food additive and colorant (E100) [12]. However, as elaborated above, purified nCUR is always contaminated by other chemicals (e.g., demethoxycurcumin, bisdemethoxycurcumin, oils and resins) [12], which may or may not influence the biological effects attributed to nCUR. A large number of CUR analogs have thus been synthesized in recent years, and many displayed activities similar to the mother compound [12,13]. A more recent approach, fully synthetic manufacturing of diferuloylmethane (sCUR), offers the potential to study the effects of this particular molecule without interference by contaminants.

In our line of work, i.e., the characterization of CUR as a potential therapeutic agent against cancer therapy-induced oral mucositis [4,5], sCUR offers a number of potential advantages. In contrast to turmeric with its musky, bitter, and piquant flavor [12], it is entirely odorless and tasteless, and therefore suitable as an ingredient of an orally administered preparation, particularly for children. Since it appears somewhat more soluble in DMSO than nCUR, aqueous solutions for oral application can be produced with less DMSO (i.e., 0.04% vs. 0.1%, vol/vol). In this context, it is worth mentioning that CUR has recently also been shown to inhibit adherence to teeth and extracellular matrix of *Streptococcus mutans*, the major causative agent of human caries [14,15]. In the present study we found that antimicrobial activity of sCUR in standard time-kill analyses was indistinguishable from nCUR [5,16,17] on a weight per volume basis for 3 major pharyngeal species (Figure 2).

With respect to prevention of mucositis, the key findings of this comparative analysis addressed the anti-inflammatory properties of the two different CUR preparations and are shown in Figures 3, 4, 5, and 6. First, both nCUR [4] and sCUR inhibited bacterial adherence to pharyngeal epithelial cells to a similar extent. Attachment of microorganisms is the first step in initiating inflammation by avoiding their elimination by the flow of mucosal secretions. It enhances their ability to trap nutrients and their rate of multiplication [18]. Attachment also allows intimate interaction with cellular pattern recognition receptors (e.g., Toll-like receptors), which activate downstream pro-inflammatory cascades including activation of NF-κB [18,19]. Second, there was no difference in the invasion ratio of live bacteria exposed to either nCUR or sCUR. However, in contrast to previous findings [4], we observed no CUR-induced inhibition of cellular penetration. Third, both nCUR and sCUR equally inhibited the secretion of several pro-inflammatory cytokines (e.g., IL-8, IL-6, MCP-1, GM-CSF) upon stimulation with live bacteria. This finding provides indirect evidence that nCUR and sCUR are equally potent in inhibiting the dissociation of IκB from NF-κB [20], and thus in down-regulating inflammation. To our knowledge, this is the first study, which compares sCUR and nCUR in an *in vitro* model designed to mimic the pro-inflammatory action of bacteria on human pharyngeal cells.

Our study has a number of limitations. We used a simple mucositis model, which may substantially deviate from true *in vivo* conditions. Also, CUR is reported to be more stable at pH values below 6.0 [12]. We used solvents with a buffered pH around 7.0. We consider this justified because human salivary pH typically varies between 6.5 and 7.2. Our data are thus relevant for studies mimicking the topical use of CUR in the oral cavity.

## Conclusions

The data provided here demonstrate that sCUR and nCUR are equivalent in a number of *in vitro* biological assays, which are designed to mimic bacteria-induced mucosal surface inflammation. The fact that the concentrations used are far below the daily allowances as a food additive for oral CUR (E100), which amounts to 3 mg/kg/d of nCUR, warrant the claim that sCUR used at a concentration of 200 μM (e.g., 10 ml of mouth rinse 4 times daily amounts to a total daily dose of 2.94 mg) is clearly in the range declared safe by the European Food Safety Authority [21]. An additional advantage in manufacturing sCUR is the fact that there is no batch-to-batch variation in the CUR content. Thus, sCUR appears to be a safe, equipotent and more palatable alternative to nCUR, and an excellent candidate for topical use in clinical oral mucositis trials.

## Competing interests

The authors declare that they have no competing interests.

## Authors’ contributions

SL conceived the study, participated in its design and drafted the manuscript. JG participated in the study design, provided pharmaceutical input, and was responsible for HPLC studies. RT participated in the study design and carried out all experimental studies. CA participated in the design of the study, drafted the experimental protocols and helped to draft the manuscript. All authors’ read and approved the final manuscript.

## Pre-publication history

The pre-publication history for this paper can be accessed here:

http://www.biomedcentral.com/1472-6882/14/53/prepub
